# Watermelon in Dysentery

**Published:** 1869-10

**Authors:** 


***Watermelon in Dysentery.***

CUCUMBERS have always had a bad reputation in "bowel complaints," and watermelons, cantelopes, etc., have been almost equally liable to criticism. But recent correspondents of the

*Boston Medical and Surgical Journal* boldly report cases of recovery after ingestion of these much-abused vegetables. Whereupon the witty editor of the *New York Medical Gazette* writes: "If this sort of thing goes on, we shall expect good results from a diet of fried pork, lobster salad, and hot plum-cake in enteric troubles generally." To which, in all seriousness, we reply, worse treatment has happened. In the late war many of the most obstinate "enteric" cases recovered rapidly when allowed unrestricted indulgence of their appetites in the cook-room. Raw onions, turnips, carrots, green corn, and half-ripe fruits, even, were often found salutary to the soldiery. If it is objected that the vegetable diet was better borne on account of the scorbutic diathesis usually present in these cases, we reply, scorbutus is but a name, and a similar condition is often produced in civil life by mistaken habits based on mistaken physiological notions. Nothing is more frequently met than the wonder of patients, or their parents, that they should have diarrhœas or dysenteries when they have so carefully abstained from "fruits and green things." During epidemics of cholera, diarrhœa or dysentery, this scrupulous caution is more likely to be carried to excess, and our experience is that the most cautious are the first to fall victims. In the progress of many long-continued diseases the system is liable to fall into similar impoverishment, from careful exclusion, from the diet table, of what are really essential ingredients.

We trust not to be misapprehended, for it is unquestionably true that when the digestive organs have not been habituated to the use of any particular kind of food, they are likely to be intolerant of it. But this no more proves that they are not needed by the system, than the fact that a starving man can not be at once permitted to satisfy his appetite, without extreme danger, proves that food is in itself injurious to him.

It is exceedingly difficult to overcome this *fruit-and-vegetable-phobia*, on the part of both physicians and patients. The simple fact is, without resorting to any very gross humoralism, a large number of these cases are caused by an effort at elimination, by the intestinal mucous-membrane, of effete and poisonous matters which the kidneys fail to excrete. These much maligned vegetables furnish pabulum to the blood, and stimuli to the kidneys, very often beyond the power of any so-called medicine to imitate.

The watermelon and cucumber cures need astonish no one. The great trouble is, we shall have watermelon and cucumber curers of dysentery and diarrhœa who will speedily kill some people whose dysenteries or diarrhœas have quite a different pathology, and then the vegetables, like many, sometimes, ambitious and vaunted members of the Materia Medica, will go away into deep disgrace. The proper relation of food and force being, by and by, better understood, we may hope to combat disease by such agencies only as nature employs in building up the human body.

***Professional Perspicuity--Again.***

In view of the indignation aroused by a certain editorial criticism, headed "Professional Perspicuity," in the August number of the Journal, upon a report of an *alleged* case of tetanus, contained in the July number of the *Chicago Medical Examiner*, we desire to explain, for the benefit of some who appear not to appreciate the fact, that a medical journal is designed to be, not a receptacle for literary rubbish, nor an advertising medium for the elevation of individuals into notoriety, but a source of *reliable* information to the profession; a record of *facts;* an index of scientific progress; and a medium for sifting the wheat from the *chaff*, the true from the *false*, in the current professional literature of the day. To the accomplishment of this object all our efforts shall be directed, and to that end we invite from *professional gentlemen* desiring to co-operate in the work, contributions of their views and opinions upon such topics as may interest the profession at large, whether in the shape of essays, or reports of cases which have *actually occurred under their observation*, reserving to ourselves the right to reject all which may, either from lack of scientific or practical interest, from *unreliability* of authorship, from *improbability* of incident, or from manifest *fraud*, seem to us to merit a place in the waste basket. To communications coming under the latter category, whether reaching us directly, or through the media of other journals, we shall show no mercy, and their authors need expect none. To all such who now, or hereafter may consider themselves aggrieved by legitimate criticism, we say, keep out of print; do not endeavor to exchange respectable obscurity for disreputable notoriety. W. H.

### *Hospital Reports.*

The attention of our readers is especially directed to the Hospital Reports contained in the present number. Each number of the Journal, hereafter, will contain a condensed summary of every thing interesting which has occurred in the hospitals of Chicago during the preceding month. These reports will be prepared under the immediate supervision of gentlemen of the professional staff of each hospital, respectively, and the profession at large will find in them an invaluable source of clinical instruction, and index for practical reference.

The city of Chicago possesses hospital facilities whose magnitude and excellence have been hitherto not properly appreciated by the profession at large, and we shall make the Journal the medium for rendering this source of professional knowledge available to our readers. We here tender our acknowledgments to Dr. Fenn, of Cook County Hospital, and Dr. Owens, of St. Luke's, for valuable contributions. W. H.

### *Appointment.*

At the last meeting of the Trustees, Dr. W. W. Allport was unanimously elected Surgeon Dentist of St. Luke's Hospital.

### *Ovariatomy.*

Drs. Benson and Yandell, of Louisville, Kentucky, and Dr. Tom O. Edwards, of Lancaster, have each recently successfully performed ovariatomy.

### *Medical Portraits.*

A series of 100 photographs of eminent European medical men is offered for sale by Dr. W. H. Helm, of Sing Sing, N. Y. The likenesses are excellent, and are sent by mail at the low price of $15 for the 100, or $2 per dozen. They can probably be procured in this city of W. B. Keen & Cooke.

### *Our Home Physician.*

We have received specimen pages of a new work with this title (by George M. Beard, M.D., of New York, assisted by Professors D. B. St. John Roosa and B. Howard), intended as a popular treatise on medical practice, with the praiseworthy object of displacing the various empirical "Guides to Health," etc., etc., with which the country abounds.

From a glance at the pages sent us, we should judge it well adapted for the purpose, but we are unwilling to fully indorse it without examining the work as a whole. We are unwilling to forestall either our own or the public's opinion. The question of the real value of such books is still an open one. We believe that the best way for all concerned is to popularize knowledge of *scientific* physiology, and displace the quack varieties thereof, which every where abound.

